# The Early Nutritional Environment of Mice Determines the Capacity for Adipose Tissue Expansion by Modulating Genes of Caveolae Structure

**DOI:** 10.1371/journal.pone.0011015

**Published:** 2010-06-21

**Authors:** Leslie P. Kozak, Susan Newman, Pei-Min Chao, Tamra Mendoza, Robert A. Koza

**Affiliations:** 1 Pennington Biomedical Research Center, Baton Rouge, Louisiana, United States of America; 2 Department of Nutrition, China Medical University, Taichung, Taiwan; Institute of Preventive Medicine, Denmark

## Abstract

While the phenomenon linking the early nutritional environment to disease susceptibility exists in many mammalian species, the underlying mechanisms are unknown. We hypothesized that nutritional programming is a variable quantitative state of gene expression, fixed by the state of energy balance in the neonate, that waxes and wanes in the adult animal in response to changes in energy balance. We tested this hypothesis with an experiment, based upon global gene expression, to identify networks of genes in which expression patterns in inguinal fat of mice have been altered by the nutritional environment during early post-natal development. The effects of over- and under-nutrition on adiposity and gene expression phenotypes were assessed at 5, 10, 21 days of age and in adult C57Bl/6J mice fed chow followed by high fat diet for 8 weeks. Under-nutrition severely suppressed plasma insulin and leptin during lactation and diet-induced obesity in adult mice, whereas over-nourished mice were phenotypically indistinguishable from those on a control diet. Food intake was not affected by under- or over-nutrition. Microarray gene expression data revealed a major class of genes encoding proteins of the caveolae and cytoskeleton, including *Cav1*, *Cav2*, *Ptrf* (Cavin1), *Ldlr*, *Vldlr* and *Mest*, that were highly associated with adipose tissue expansion in 10 day-old mice during the dynamic phase of inguinal fat development and in adult animals exposed to an obesogenic environment. In conclusion gene expression profiles, fat mass and adipocyte size in 10 day old mice predicted similar phenotypes in adult mice with variable diet-induced obesity. These results are supported by phenotypes of KO mice and suggest that when an animal enters a state of positive energy balance adipose tissue expansion is initiated by coordinate changes in mRNA levels for proteins required for modulating the structure of the caveolae to maximize the capacity of the adipocyte for lipid storage.

## Introduction

While genetic variation among individuals is a major contributing factor to the development of obesity [1]–[3], it has been argued that genetic variation by itself cannot account for the obesity epidemic; but rather, an environment, increasingly conducive to the development of obesity, has become a second major contributor [4]–[6]. A realization of the importance of early nutrition on susceptibility to obesity initially emerged from an epidemiological study of adult men who were at critical stages of fetal or post-natal development during the Dutch Famine in the winter of 1945 [7]. Under-nutrition during late gestation and early post-natal development led to adults who were resistant to the development of obesity. On the other hand, undernourishment of the developing fetus caused them to be under weight at birth and those individuals experiencing catch up growth during early post-natal development had increased susceptibility to type 2 diabetes. Early malnutrition also affects susceptibility to cardiovascular disease and hypertension [8]–[11] and has led to the Barker hypothesis of fetal nutritional programming. There is much evidence that the nutritional environment of the developing mammal has an impact on long term development of organ systems associated with energy metabolism, however, the molecular mechanisms by which early nutrition affects the expression of genes critical for organ system phenotypes, such as the capacity to expand adipose tissue mass is almost completely unknown.

The effects of the early nutritional environment on adiposity in an animal model were first described in 1962 by McCance [12], and later by others [13], [14] who showed that suckling rats and mice with restricted access to food were smaller and never caught up to those with unlimited food availability. These studies suggest that similar obesity phenotypes in animal models and humans are caused by the dynamics of competitive feeding, growth and dominance behaviors during early post-natal development. The capacity of an animal to expand its adipose tissue in an obesogenic environment is a vital factor in determining the progression of the animal towards pathology, in particular the metabolic syndrome [15]. In its simplest form adipose tissue expansion is determined by both variation in the hypertrophy of existing adipocytes and hyperplasia of preadipocytes from a stem cell population [16]. The identification and properties of white adipose tissue progenitors in the stromal-vascular fraction of adipose tissue has recently become a topic of intense investigation [17], [18]. Still little is known as to when and how these progenitors can be recruited in a state of positive energy balance [19]. Similarly, while decades of studying the fundamentals of the adipogenic process in 3T3-L1 preadipocytes in tissue culture has led to descriptions of cell proliferation and of the transcription and signaling pathways of adipogenesis [20], the basis for adipocyte expansion in response to an obesogenic environment remains incomplete. We are using an experimental strategy to evaluate the effects of gene-environmental interactions on obesity at the molecular level that is based upon the unexpectedly large variation in diet-induced obesity (DIO) among genetically identical C57BL/6J (B6) mice [21]. Thus, the effects of a variable environment can be evaluated independent of genetic variation. Variation in DIO among B6 mice is a stable phenotype that affects all white fat depots, it is highly correlated with adipocyte size and with elevated expression of several novel biomarkers that provide an opportunity to study molecular mechanisms [21], [22].

A gene with the strongest association to adipose tissue expansion is the imprinted gene *Mest*. *Mest* expression, which is expressed almost exclusively in adipose tissues of the mouse, is highly correlated with DIO in adult mice; its activity at 7 weeks of age before the introduction of a high fat diet is also correlated with susceptibility to DIO later in life; it is not induced by a diet high in saturated fat, unlike *Scd1*; its expression during early postnatal development closely tracks with the developmental profile of fat mass expansion and *Mest* mRNA is highly correlated with the MEST protein [21], [22]. As far as adipose tissue is concerned, *Mest* may be more highly correlated with fat mass expansion than possibly any other gene so far analyzed, exceeding even that of leptin (this study). MEST is localized to the endoplasmic reticulum/Golgi apparatus where its putative enzymatic properties as a lipase or acyltransferase, predicted from sequence homology with members of the _α/β_fold hydrolase superfamily, can enable it to function in lipid accumulation under conditions of positive energy balance [22]. However, the precise biochemical function of MEST in the expansion of fat mass is still unknown. Since it is unlikely that MEST functions alone in fat mass expansion, we have designed an experiment in which fat mass expansion is manipulated as a consequence of developmental age and the nutritional environment. Since we know *a priori* how MEST is expressed under these conditions, identifying genes that are highly correlated with *Mest* will provide a link to other genes primarily involved in adipose tissue expansion.

An important corollary to determining MEST function in adipose tissue expansion is that variation in diet-induced obesity in genetically identical adult mice could have developmental origins. We observed that mice destined to gain the most weight could be identified from their elevated levels of adiposity and expression of *Mest*, *Bmp3* and *Sfrp5* in adipose tissue at 7 weeks of age before they were fed a high fat diet [21]. Accordingly, this evidence that susceptibility to obesity arose from variation in the early nutritional environment led us to hypothesize that expression levels of MEST-associated gene networks, which control adipose tissue expansion, become partially set during the first weeks of life and remain so for the life of the animal. To test this hypothesis we have raised mice under conditions of over- and under-nutrition during early post-natal development to establish their adiposity phenotypes from early development to adulthood. Microarray analysis was then conducted to obtain a global perspective of gene expression in inguinal fat during both early development and in adult mice in an obesogenic environment. Venn analysis revealed that the major class of genes associated with adipose tissue expansion in both suckling mice and adult mice fed a high fat diet were those encoding elements of the caveolae, cytoskeleton and lipid vesicle structure. Correlation analyses suggest that gene expression phenotypes established in individual mice during early development are re-expressed in adult mice. These novel findings show that the nutritional environment during early post-natal development controls the capacity of the adipocyte to accumulate fat by modulating the expression of a subset of genes associated with the structure of caveolae and that this capacity for structural remodeling is recapitulated in the adult mouse depending upon the nutritional environment.

## Results

### Effects of over- and under-nutrition on adiposity

**Figure 1:**
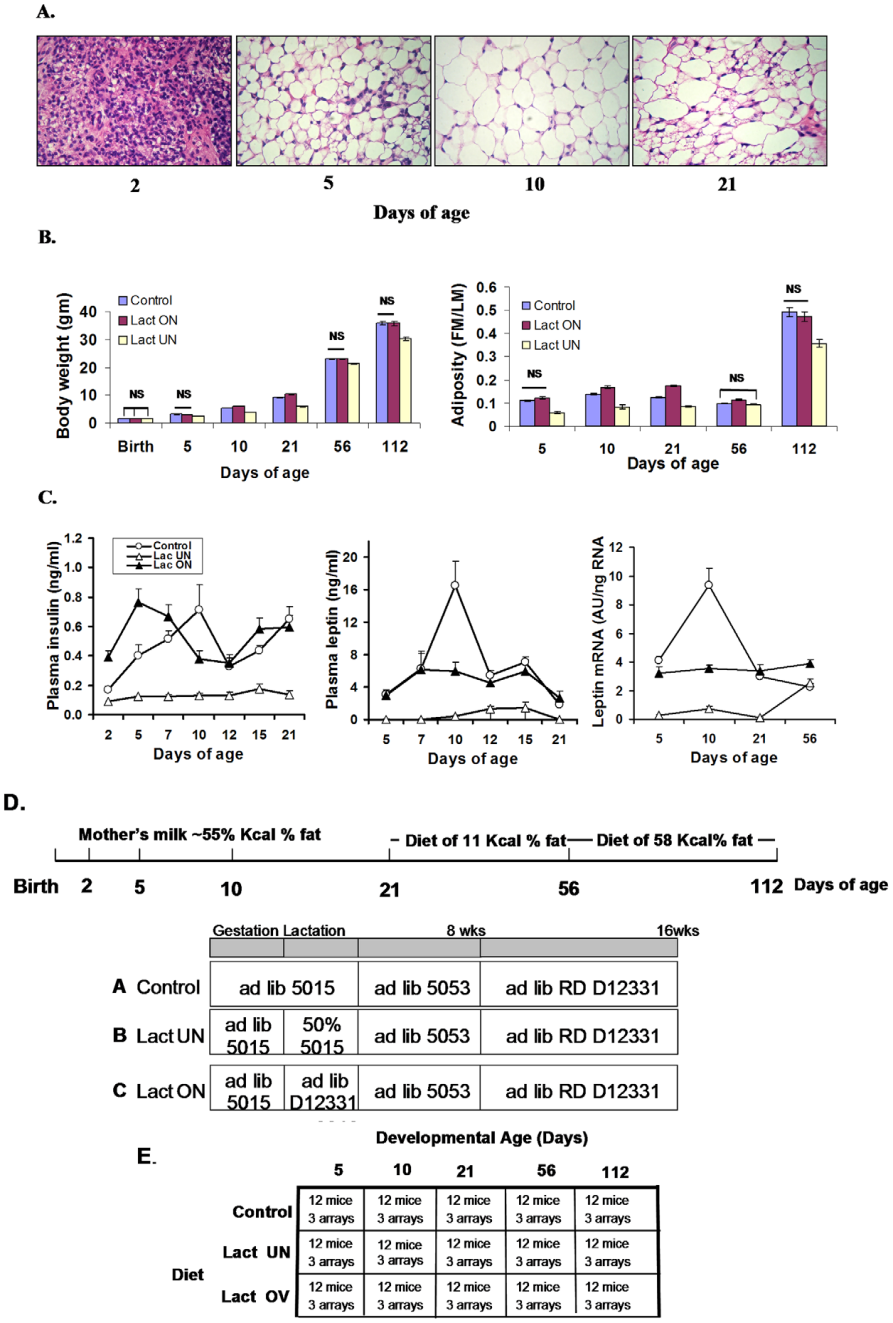
Phenotypes of adiposity in B6 mice during variation in the early nutritional environment. **A**. Morphology of inguinal fat between 2 and 21 days of age as revealed by hematoxylin-eosin staining of paraffin-embedded tissues; magnification was 20×. **B**. Effects of under-nutrition (LUN) and over-nutrition (LON) from birth to weaning at 21 days of age on body weight and adiposity index (FM/LM). Statistical analysis to determine significant differences between Control vs LON and Control vs LUN was done with a 2-tailed t-test. Except where indicated groups were different at P<0.001; the numbers of animals ranged from 36 to 80. Detailed data on a subgroup of these mice is provided in Supplemental Table S1. **C**. Effects of LUN and LON on plasma insulin and leptin were determined by Elisa assays. Leptin mRNA levels in the inguinal fat depot from mice 2 to 21 days of age were determined by qRT-PCR. The number of mice in each group equaled 12. Using leptin mRNA and protein data for individual mice from the three nutritional groups between 2 and 21 days of age, the correlation coefficient (R) between plasma leptin and leptin mRNA in the inguinal fat depot was 0.894. D. A schematic diagram of the nutritional protocol from birth to 112 days of age. E. A matrix describing the groups analyzed for gene expression by microarray analysis. RNA was isolated and purified from the inguinal fat depot from individual mice and equal aliquots from each mouse used to construct a pool. Three microarrays from each pool was analyzed.

The design of the nutritional experiment, as shown in Fig. 1D, aims to perturb adiposity and patterns of gene expression in the inguinal fat depot in control mice (Control) by under-nutrition (LUN) and by over-nutrition (LON) during lactation (birth to 3 weeks of age). Adiposity phenotypes were determined and plasma and tissue for protein and mRNA analysis were collected from: 1.) suckling mice at 5 and 10 days age, 2.) 21 day old mice at weaning, 3.) 56 day-old adult mice fed a low fat chow diet for 5 weeks, and 4.) 112 day old adult mice fed a high fat diet for 8 weeks. At 5, 10 and 21 days of age mice directly experienced the 3 dietary conditions, whereas between 21 and 56 days all mice were fed a low fat diet and from 56 to 112 days of age a high fat-high sucrose diet. Three cohorts of mice followed this protocol: Cohort I (180 mice) generated 12 mice for each age and nutritional treatment that were sacrificed for plasma and inguinal fat for microarray and qRT-PCR analysis of gene expression; Cohort II (135 mice) establish adipose phenotypes for each nutritional group from 5 days until 112 days of age. At 112 days of age, mice were sacrificed to isolated RNA from inguinal fat from gene expression analysis; Cohort III (249 total mice, 119 males) raised under control conditions only, were measured for adiposity at 5 and 10 days of age and were sacrificed at 10 days of age to provide RNA for gene expression. Cohort III was designed to recapitulate our experiment in which adult mice raised under control conditions are fed a high fat diet from 56 to 112 days of age to induce DIO that was highly variable and showed high correlations to the expression of *Mest* and *Bmp3*. All adipose tissue analyses including histological and gene expression with microarrays and qRT-PCR were performed with the inguinal fat depot because it is the only macroscopically visible white fat depot in the mouse from about 1–2 day of age, when it first becomes detectable, until about 9 days of age when the visceral fat depots begin to emerge. Thus, during the most critical time of suckling when the pups first independently experience a dynamic nutritional environment, the inguinal fat depot is “the white fat depot” by which we can monitor changes in the expression of genes related to energy balance.

The effects of the nutritional protocols on adipose tissue morphology, body weight, adiposity (fat mass/lean mass; FM/LM), the levels of plasma insulin and leptin and leptin mRNA in inguinal fat depots are shown in Fig 1 and Table 1. The inguinal fat depot of a 2 day-old control mouse has very little fat deposited in its adipocytes, by 5 days of age most adipocytes are partially filled with lipid that increases even more in 10 day old mice (Fig. 1A). However, consistent with previous reports, at 21 days of age the mature white adipocyte population is heterogeneous in size and distinguished by the transient appearance of brown adipocytes [23]. These changes in histology are reflected in changes in body weight and adiposity index (Fig. 1B). Of particular note is the modest increase in adiposity index from 5 to 10 days of age that was unchanged at 21 days of age; it subsequently declines to its lowest values at 56 days of age and then increases to high levels following 8 weeks on a high fat diet. Thus, while the high adiposity index in 10 day old mice reflects the consumption of high fat milk during suckling, it does not reach the high levels found in adult mice with diet induced obesity, even in LON mice. From both the histology and measurements of adiposity, under-nutrition did not seem to affect the ability of LUN mice to initiate the accumulation of lipid in adipocytes, the fat depots of both control and LUN mice had very little lipid accumulated at 2 days of age and a major increase occurred by 5 days of age. However, from 5 days of age onward the LUN mice simply had smaller adipocytes indicative of reduced lipid accumulation (data not shown). At the histological level here were no detectable differences between Control and LON mice. The absence of effects on adiposity between control and LON mice were quantified by determinations of body weight and composition as shown in Fig. 1B and Table 1. We estimate that the fat content of the milk consumed by a 5 day old mouse will contribute no more than 7.5 to 10% of fat detected by NMR. Throughout the entire experiment from 5 days of age until 112 days of age significant differences in adiposity index in Control mice compared to LON mice of 17%, 30% and 15% were detected only at 10, 21 and 56 days of age, respectively (Fig. 1B). Control and LON protocols did not differ in their long-term effects on DIO. On other hand, the LUN mice had significantly reduced body weight, fat mass and lean mass at every age and even at 56 days of age after all mice had been fed the same low fat chow diet for 5 weeks (Fig. 1B and Table 1). At 112 days of age after mice were fed a high fat diet for 8 weeks, the increase in adiposity in the LUN mice was also much lower than in Control and LON mice (Fig. 1B).

**Table 1:** Adiposity phenotypes among groups of mice submitted to control, under-nutrition and over-nutrition dietary conditions from birth to weaning.

		Nutritional Conditions from Birth to Weaning (21 days of age)					
		Control (n = 44)		Under-Nutrition (n = 26)		Over-Nutrition (n = 43)	
		Mean (gm)	Std. Dev.	Mean (gm)	Std. Dev.	Mean (gm)	Std. Dev.
Birth	Body weight	1.35^a^	0.1	1.39^a^	0.08	1.37^a^	0.11
Day 5	Body weight	3.16^a^	0.4	2.4^b^	0.3	2.95^a^	0.43
	Fat Mass	0.31^a^	0.08	0.11^b^	0.05	0.32^a^	0.12
	Lean Mass	2.75^a^	0.39	2.18^b^	0.38	2.55^a^	0.34
Day 10	Body Weight	5.53^a^	0.71	3.79^b^	0.46	5.71^a^	1.01
	Fat Mass	0.69^a^	0.2	0.28^b^	0.11	0.83^a^	0.31
	Lean Mass	4.77^a^	0.46	3.56^b^	0.36	4.47^a^	0.63
Day 21	Body Weight	9.67^a^	1.08	6.45^b^	1.16	10.09^a^	1.25
	Fat Mass	1.06^a^	0.261	0.57^b^	0.2	1.41^c^	0.32
	Lean Mass	8.06^a^	0.86	5.56^b^	0.81	8.21^a^	0.91
Day 56	Body Weight	22.96^a^	1.39	21.5^b^	1.24	22. 5^a^	1.52
	Fat Mass	1.75^a^	0.44	1.51^b^	0.27	2.04^c^	0.38
	Lean Mass	17.95^a^	1.36	16.9^b^	1.18	17.7^ab^	1.32
Day 112	Body Weight	35.95^a^	4.2	30.7^b^	2.78	35.48^a^	4.42
	Fat Mass	10.92^a^	3.23	7.18^b^	2.13	10.36^a^	3.43
	Lean Mass	21.99^a^	1.31	20.5^b^	0.85	21.68^a^	1.43
Daily Food Intake (gm)		2.39^a^	0.19	2.25^a^	0.25	2.12^a^	0.21
Leptin (ng/ml) Day 10		7.24^a^	5.75	0.66^b^	2.42	8.51^a^	10.15
Day 112		24.80^a^	7.91	12.48^b^	7.58	20.60^a^	10.01
Mest (AU/ng RNA) Day 112		40.76^a^	26.05	20.10^b^	14.50	34.88^a^	31.73

Each group was then fed the same low fat chow diet from weaning to 8 weeks of age and then a high fat diet for an additional 8 weeks. Values with the same superscript letter are not significantly different from each other. The level of significance was set at P<0.01. N for leptin analyses = 24. Food intake was assessed from day 98 to 112.

The nutritional protocol of LON caused a precocious hyper-insulinemia similar to rat pups fed a high carbohydrate diet (Fig. 1C) [24]. The profile of plasma leptin in control mice was similar to that previously shown [25], [26] (Fig. 1C). LUN mice had severely suppressed plasma insulin and leptin levels. At 10 days of age LUN mice were hypoglycemic with non-fasted blood glucose levels of approximately 60 mg%, compared to mice on a control diet (100 mg%). Although plasma leptin at 10 days of age was somewhat higher in the Controls than in the LON mice (Fig. 1C), an analysis on a larger cohort of mice at 10 days of age did not show a significant difference between Control and LON mice (see Table 1). The variance in plasma leptin levels at this early age was very high making it difficult to establish significant differences. A very high correlation was found between the levels of plasma leptin and its mRNA levels in the inguinal fat depots at 10 days of age (R = 0.894). Similar correlations were previously found in adult mice [27], [28].

### Global gene expression in the developing adipose tissue during malnutrition

Based upon the effects of the early nutritional environment on adiposity, as shown in Fig. 1 and Table 1, we have carried out an extensive analysis to identify genes with critical roles in the control of adipose tissue expansion. The design of the experiment is shown in Fig. 1E. For the total experiment comprising 15 microarray groups, each composed of a pool of 12 mice, analyzed in triplicate, 4649 quantile-normalized genes were selected for further analysis. Criteria for inclusion in the list of 4649 genes were 1.) a minimal signal of 5000 detected in at least 3 of the 15 groups and 2.) a 1.6-fold variation in expression between any two groups and 3.) a significance level (2-tailed T-Test) for the differences in expression at P<0.01 for at least 1 comparison.

**Figure 2:**
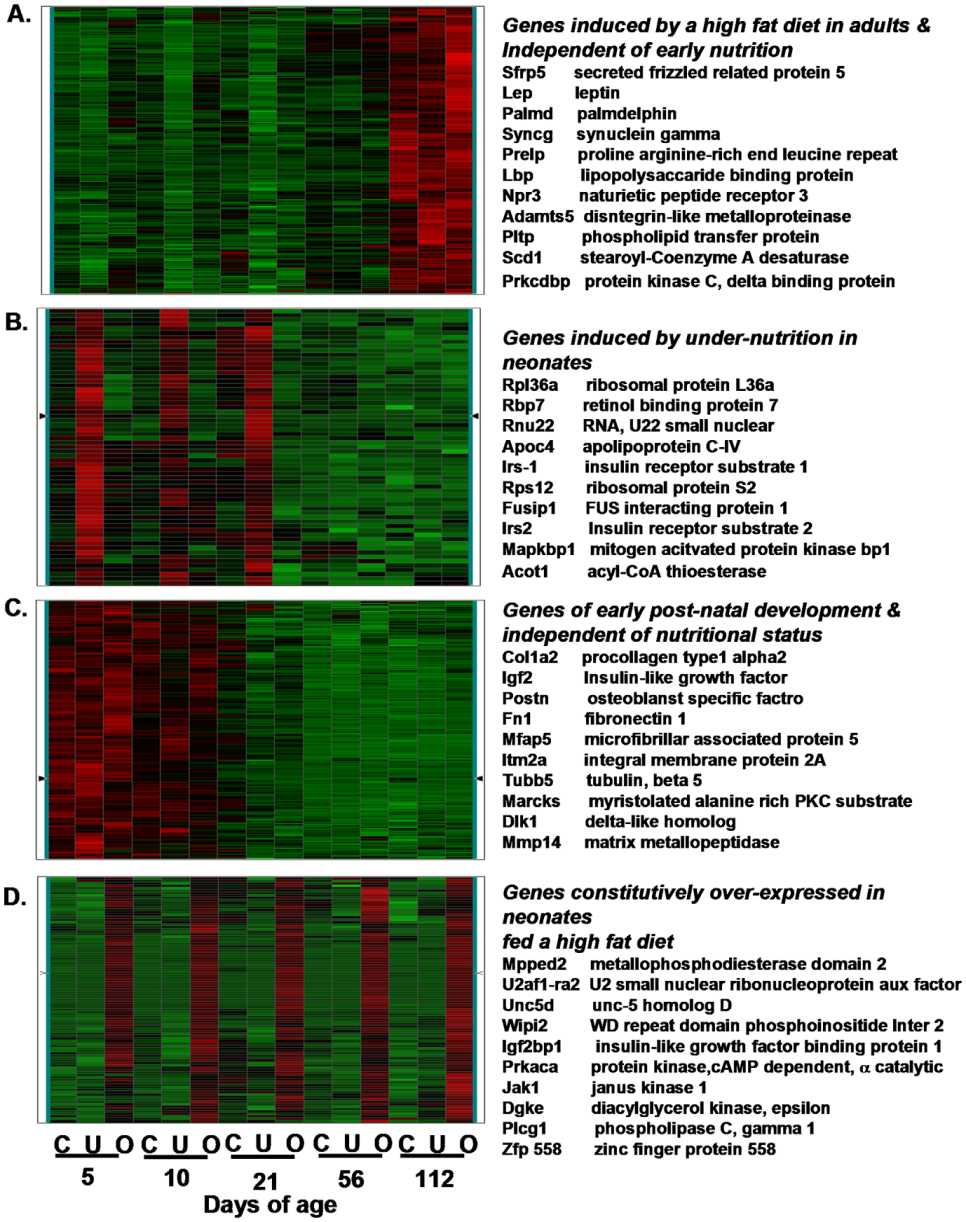
Heat maps of selective K-means clusters. Microarrays were performed as described in Materials and Methods. The nutritional conditions and age on the mice for each microarray is shown at the bottom of the figure; C represents control mice, U, under-nutrition during lactation and O, over-nutrition during lactation. Selected genes, based simply on a potential interest on the interaction of the nutritional environment and gene expression for each of 4 clusters are listed on the right-hand side of each heat map.

K-means cluster analysis generated 50 clusters composed of between 75 and 250 genes. Four clusters of gene expression are shown in Figure 2 to illustrate profiles of expression that are dependent or independent of developmental age and/or the nutritional environment. Cluster A contains genes that showed very low expression until the adult mice were fed a high fat diet at 56 days of age, whereupon expression was induced in a manner independent of their nutritional environment during suckling. Cluster B shows genes that were strongly induced in LUN mice compared to Control and LON groups during the suckling period, suppressed in 8 week-old mice on a low fat chow diet and showed no induction during the 8 weeks on a high fat diet. The genes in Cluster C were strongly up-regulated during early lactation in a manner independent of the nutritional environment and then down regulated after weaning. These could be genes with primary functions during early development that are not affected by the nutritional environment. Finally Cluster D shows a battery of genes up-regulated in LON in a manner that suggests they become constitutively up-regulated by the high fat diet fed to the lactating mother. However, despite robust induction in gene expression in suckling mice from reduced litter sizes (4 vs 8 pups) whose mothers were fed a high fat diet, this elevated gene expression did not lead to differences in adiposity compared to offspring from mothers fed the Control diet (See Fig. 1B and Table 1). The subset of genes, listed for each of the gene clusters, represents genes of interest to adipose tissue biology.

**Figure 3:**
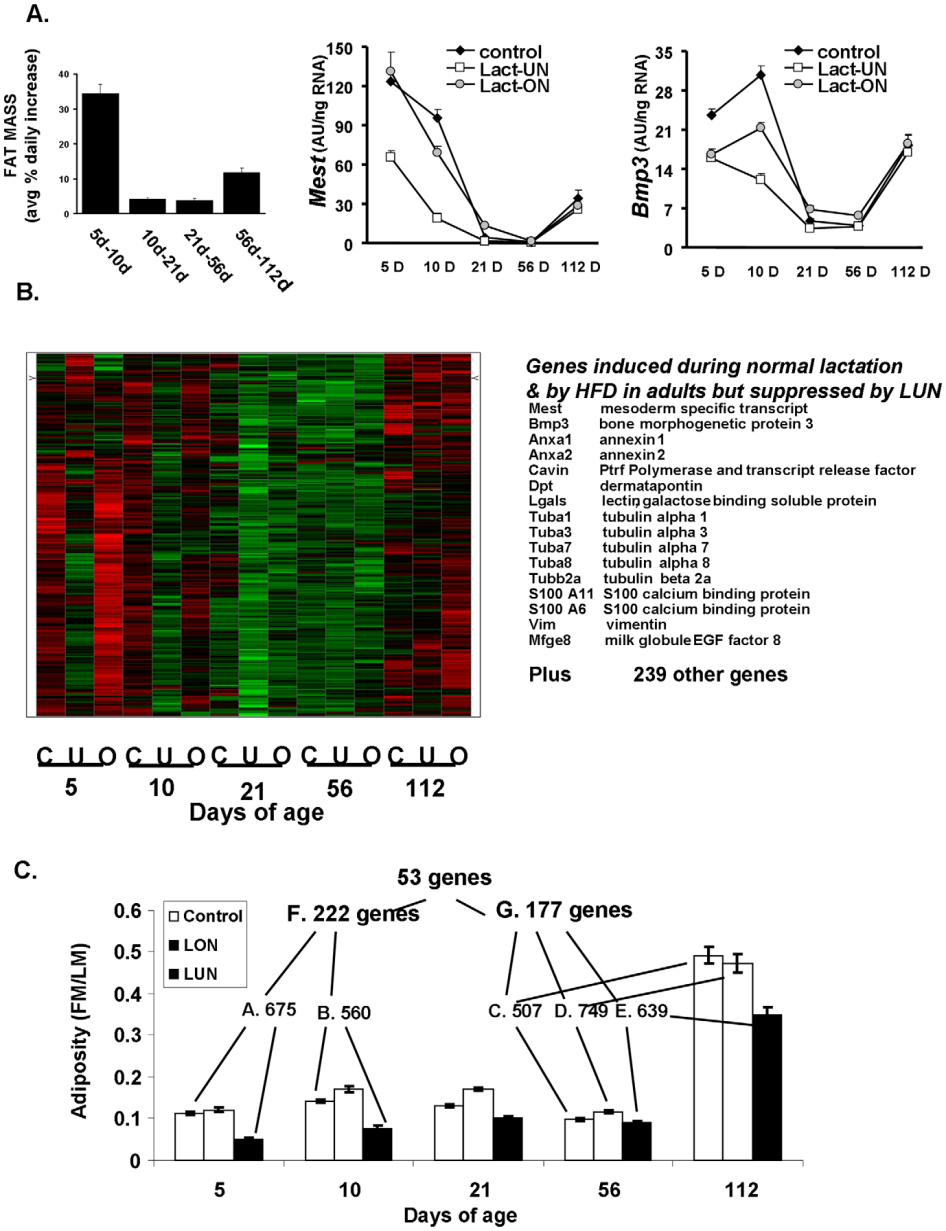
K-means cluster and Venn analysis strategies for analyzing microarray data to identify genes associated with adipose tissue expansion. **A**. The rate of percent fat accumulation per day and the levels of Mest and *Bmp3* mRNA, which have previously been shown to always correlate with increased adipose tissue expansion [21], [22], provide a pattern of gene expression as a function of developmental age and the nutritional environment that is highly correlated with adipose tissue expansion. **B**. The heat map of K-means clusters for genes resembling *Mest* and *Bmp3* and a list of candidate genes for functions related to adipose tissue expansion. C. Venn analysis to identify ATE genes up-regulated in mice as neonates during malnutrition or in adults in an obesogenic environment. The Venn analysis was designed to filter out changes in gene expression related to the effects of a high fat diet independent of adipose tissue expansion i.e. Groups C, D. E., the nutritional history or the effects of early development Groups A and B.

The variation in gene expression evident from this microarray experiment is very complex. As the experiment was designed, variation in gene expression was related to the effects of the nutritional environment on adipose tissue expansion; however, variation in other genes was related to biological processes associated with development, much of which is independent of the nutritional status, and still other genes are strongly induced by the high fat diet and are not associated with obesity. Although much of this varation in gene expression is extremely interesting, since it is not directly related to the question of adipose tissue expansion it will not pursued further herein. Therefore, to identify gene systems associated with mechanisms of adipose tissue expansion, we first identified a K-means cluster with a profile similar to the rate of fat accumulation during development and to the profiles of the *Mest* and *Bmp3* genes, previously shown to be very highly correlated with adipose tissue expansion [21], [22] (Fig. 3A). This cluster shows a group of genes that were up-regulated in Control and LON groups at days 5 and 10, while expression was down-regulated in the LUN mice (Fig. 3B). From weaning until 8 weeks of age while mice were fed a low fat chow diet, the expression levels of these genes remained low in all 3 nutritional groups; however, when the mice were fed a high fat diet from 8 until 16 weeks of age gene expression was again induced to higher levels and many of the genes were more highly expressed in mice from the control and LON groups than in the LUN group. In addition to *Mest* and *Bmp3*, genes in this cluster associated with the cytoskeleton of the adipocyte were highly represented; however, another 239 genes with diverse functions were also present in this cluster.

### Genes of adipose tissue expansion

**Figure 4:**
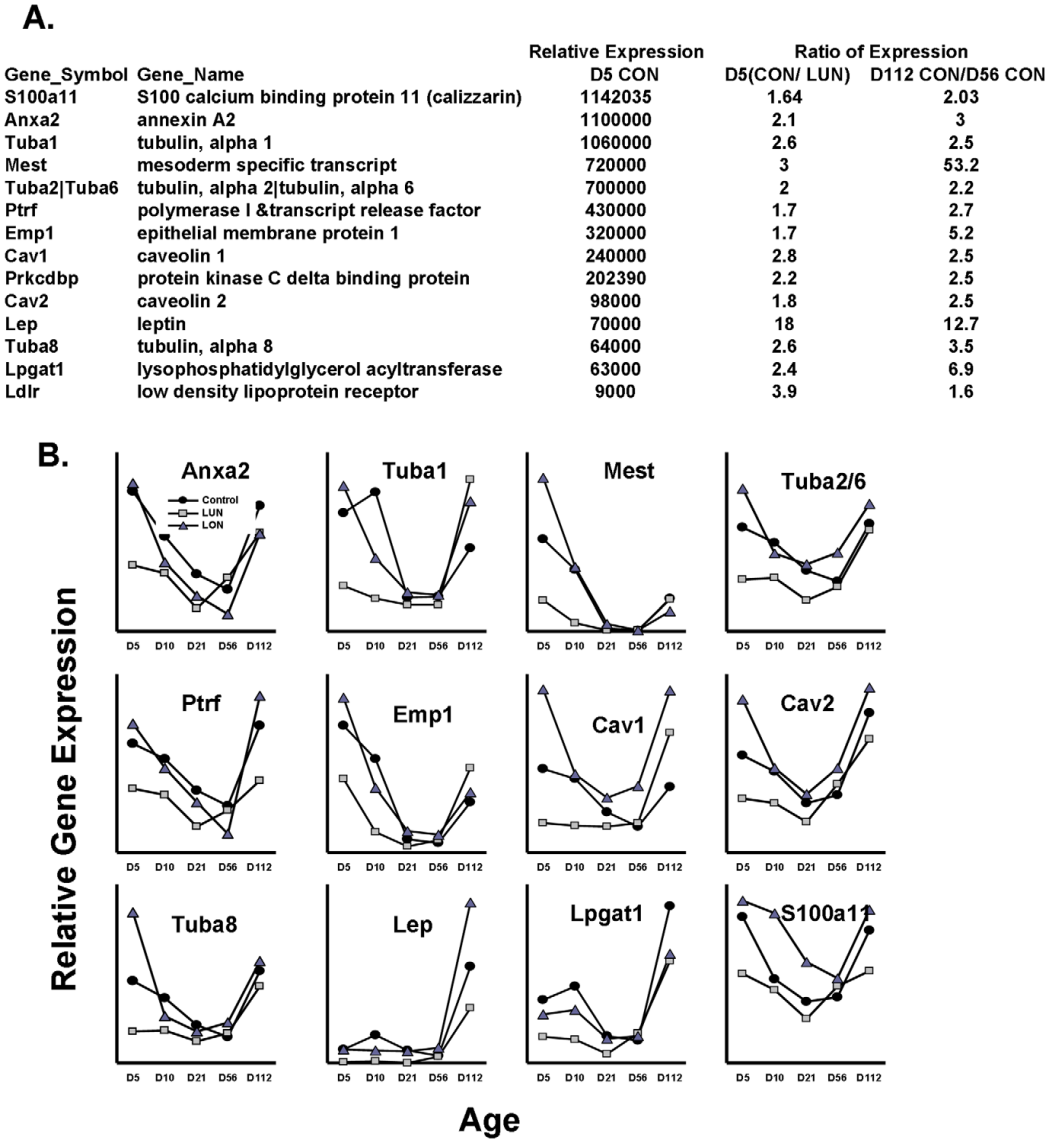
Developmental expression profiles of candidate genes of adipose tissue expansion. **A**. Top candidate ATE genes with their expression levels and ratios of induction are given on the right. **B**. The expression profile of ATE genes during development in mice raised under Control, LUN and LON conditions. Data for the levels of expression were taken directly from normalized gene expression data. Supplemental Figure S2 shows comparisons of data for several genes from microarrays with that obtained with qRT-PCR using TaqMan probes to validate the use of data from microarrays for quantitative estimates of gene expression.

K-means cluster analysis to select genes with overall profiles similar to *Mest* will still select genes that will have similar developmental profiles, but are not necessarily linked to adipose tissue expansion. To refine further the list of genes that are specifically linked to adipose tissue expansion, irrespective of the developmental age or nutritional diet of the animal, a Venn analysis approach was designed (Fig. 3C). For example, a high fat diet or caloric restriction will affect the expression of many genes that are not necessarily related to adipose tissue expansion as shown in Fig. 2C and D. In addition, developmental effects will be exerted that are also not related to expansion of adipose tissue. We designed a biological filtering strategy by Venn analysis (outlined in Figure 3C), which was driven by the morphological phenotypes shown in Fig 1B and Table 1,to remove genes not associated with adipose tissue expansion. Since very little difference in adiposity was detected between Control and LON mice, to avoid the effects of gene expression that are due to the high fat diet and not adiposity, we have only used the data for the Control and LUN groups during early post-natal development. The major variations in adipose tissue mass occurred between LUN mice and Control at 5 (Group A) and 10 days of age (Group B) and between mice at 56 days of age fed chow diet for 5 weeks and mice at 112 days of age after being fed a high fat diet for 8 weeks, irrespective of the nutritional environment during suckling (Groups C, D and E). Group F selected common genes in the neonates and Group G genes in common among adults. The final Venn analysis of Groups F and G resulted in 53 up-regulated genes (the complete list of genes uncovered by Venn analysis is given in Supplemental Table S1). A subset of 14 genes that were readily associated with a common physiological/morphological function is listed in Fig. 4A.

**Figure 5:**
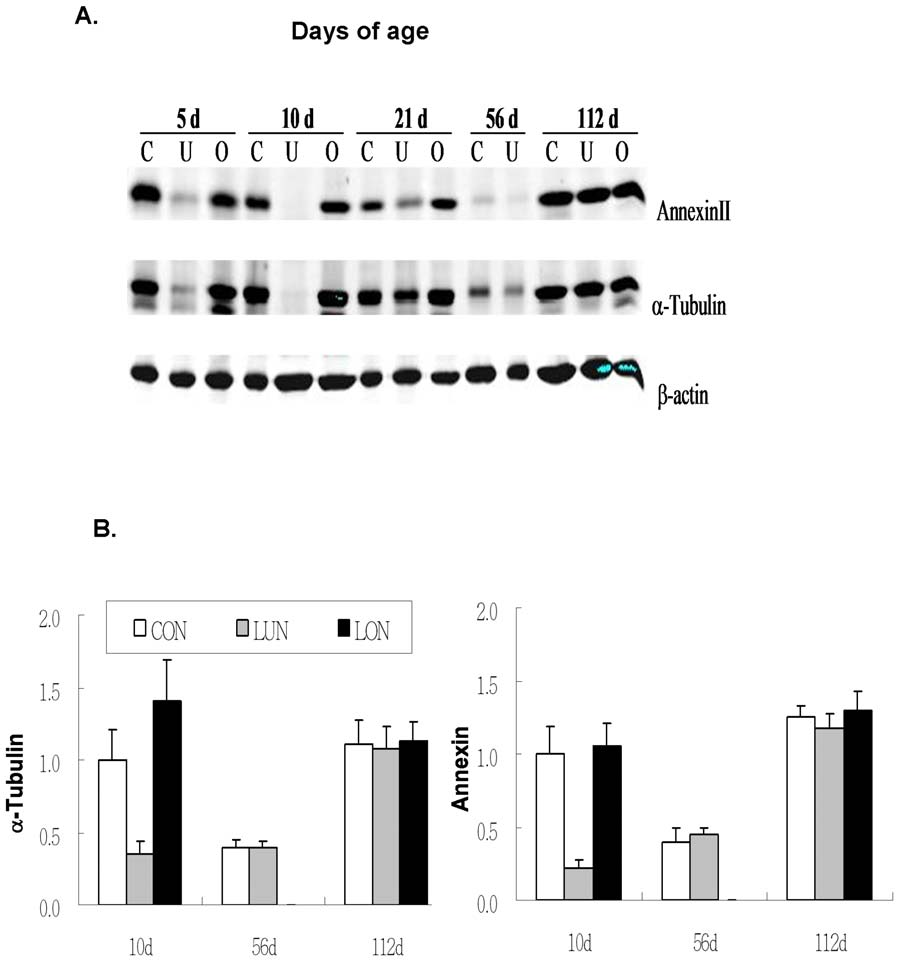
Genes encoding cytoskeletal proteins have reduced protein levels. **A**. Western blot analysis of α-tubulin and annexin A2 demonstrates that variation in gene expression corresponds to variation in the levels of protein. **B**. Relative levels of protein illustrate the dependence of protein levels on age and nutritional conditions.

**Figure 6:**
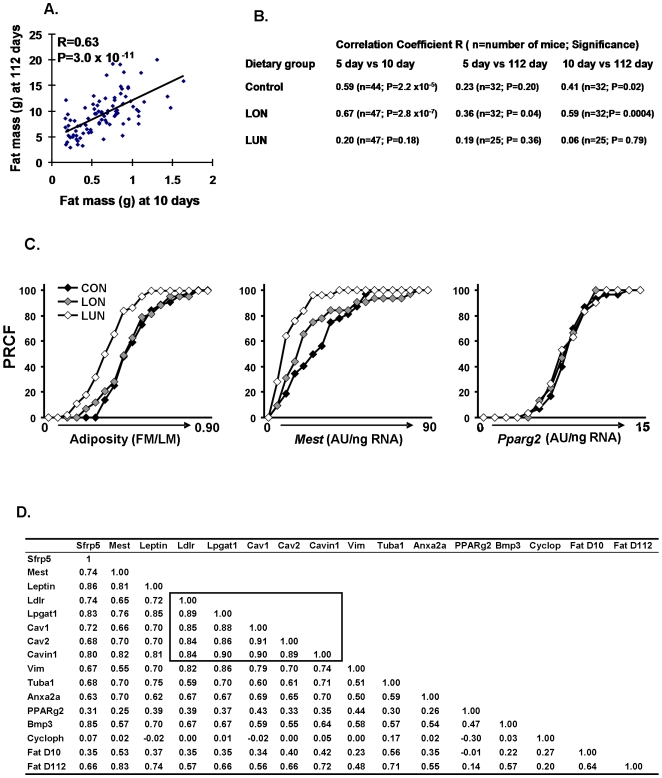
Regression analyses establishes significant associations of fat mass and ATE gene expression at 112 days of age and adiposity at 10 days of age. **A**. fat mass at 10 days of age vs fat mass at 112 days of age; **B**. Regression analyses of fat mass at 5, 10 and 11 days of age under 3 nutritional conditions show that significant associations between adiposity at 5, 10 and 112 days of age depend upon nutritional conditions that promote a positive energy balance during post-natal development. **C**. Percent relative cumulative frequency (PRCF) p the strong effects of the LUN environment on adiposity and *Mest* expression, but the lack of effects on *PPARγ* expression in 112 day-old mice fed a high fat diet for 8 weeks. Data are derived from adiposity and gene expression measurements from at least 25 mice per group. **D**. A matrix illustrating the correlation coefficients of the major ATE genes and fat mass as well as cyclophilin and PPARγ as control genes at 10 and 112 days of age; Correlations coefficients outlined by the box are unusually strong; N = 83; at R = 0.3, P = 0.01.

Similar to the K-means cluster analysis there was a high prevalence of genes associated with the cytoskeleton and membrane structures of the adipocyte related to lipid vesicle formation. Thirteen abundantly expressed genes selected from the complete list of 53 genes (Supplemental Table S1) were associated with the cytoskeleton, e. g. tubulin, vimentin (See Fig. 3B), annexins and the structure of caveolins, e. g. *Cav1*, *Cav2* and Cavin (*Ptrf*, polymerase I and transcript release factor). Some of these genes were also detected in an analysis of adipose gene expression in the presence and absence of rimonabant [29]. The expression profiles of these genes from birth to 112 days of age were remarkably similar: expression was highest immediately after birth and it was followed by a sharp decline to a nadir at 21 days of age (Fig. 4B). Levels remained low from weaning until 56 days of age while the mice were fed a low fat chow diet, then upon the introduction of a high fat diet from 56 to 112 days of age a dramatic induction of expression occurred (Fig. 4B). The expression pattern of these genes of the cytoskeleton were virtually super-imposable upon the pattern of the *Mest* gene for which increasing evidence indicates that it has a role in adipose tissue expansion [22]. In addition, the levels of all of these genes are strongly suppressed by under-nutrition during the suckling period. The suppression of annexin A2 and α1-tubulin expression in LUN mice during postnatal development was also demonstrated at the protein level (Figure 5A and B). Despite the obvious requirement for transcriptional mechanisms to modulate the changes in mRNA levels for these genes of the cytoskeleton, no unifying pattern in the expression profiles of the major genes encoding transcription factors of adipogenesis was observed (Supplemental Figure S1) and the correlation coefficients of PPARγ with fat mass at 10 and 112 days of age were non-significant (R = −0.01 and 0.14, respectively) and showed only marginal significance with some of the genes of ATE (Figure 6).

### Do adiposity phenotypes in the post-natal period predict susceptibility to diet-induced obesity in adult mice

The crux of the problem is to determine at the gene level how the early nutritional environment influences susceptibility to diet-induced obesity in the adult. To address this, we generated multiple cohorts of mice (Cohorts I and II) for regression analysis to detect associations between morphological and gene expression phenotypes as previously described. We can measure adiposity in the 112 day old adult and correlate it to gene expression in the adult because this is the end of the experiment. We can also correlate gene expression in the 112 day old mouse to adiposity in the 10 day old mouse because adiposity was measured in 10 day old mice on the way to 112 days of age. However, although we can directly correlate adiposity at 10 days of age with gene expression at 10 days of age, we cannot assay gene expression in a 10 day old mouse that will be phenotyped for obesity at 112 days of age. In our PLoS Genetics study (PLoS Genetics, 2006) we took inguinal fat biopsies at 7 weeks of age and assayed for adiposity at 112 days of age and found a significant correlation that led to this study; however, surgically removing the inguinal fat depot in a 10 day old mouse would be too traumatic.

#### A model based upon dietary manipulations of the lactating mother

Using pooled data from Control, LON and LUN groups of Cohort II, we show a highly significant correlation between fat mass at 10 days of age and 112 days of age (Figure 6A R = 0.63), suggesting that 40% of the variance in adiposity at 10 days of age is associated with adiposity at 112 days of age after being fed a high fat diet for 8 weeks. To test further the idea that the obesogenic environment during early post-natal development establishes a predisposition to obesity in adult mice, we determined the correlation coefficients between fat mass at 5, 10 and 112 days of age for each nutritional group (Figure 6B). Mice in the LUN environment showed no relationship between adiposity at 5 days of age to that at 10 or 112 days of age, whereas those raised in a control or over-nutritional environment showed highly significant associations between 5,10 and 112 days of age. The significant association (R = 0.36: P = 0.04) between adiposity at 5 and 112 days of age for the LON group was remarkable, since the morphological development of the inguinal fat at 5 days of age was still in its infancy and the visceral white fat depots were not yet evident. This data indicates that between 5 and 10 days of age in the presence of a positive energy balance, but not a negative energy balance, the capacity for adipose tissue expansion is becoming fixed and will determine the potentiality for adipose tissue expansion in adult mice in an obesogenic environment.

Of all genes selected for their association with ATE the most significant is *Mest* (Fig. 6C). Emphasis of the relationship between adiposity and gene expression phenotypes to dietary conditions is very effectively shown by graphic presentation of data as percent relative cumulative frequency to emphasize the absence of changes in PPARγ mRNA, a key regulator of adipogensis (PRFC; Fig. 6C) [30]. The shift in the curves to the left for the LUN groups in the plots for adiposity and *Mest* shows the suppression of adiposity and *Mest* expression by early post-natal malnourishment, on the other hand, expression of PPARγ is unaffected by the dietary protocol as indicated by the regression data between PPARγ and Fat at D10 and D112 (R = −0.01 and 0.14, respectively). A complete summary of the association of genes to adiposity and each other is presented in Figure 6D. At 112 days of age, expression of all ATE genes showed highly significant correlations with fat mass at 112 days, albeit none were as significant as *Mest* (R = 0.83; Fig. 6C and D). In addition, gene expression at 112 days of age was also positively correlated with fat mass at 10 days of age, though with lower R-values than fat mass at 112 days (for Mest R = 0.53; Fig. 6D). A strong associaton of *Mest* with adiposity in adult mice was previously found by our laboratory and others [21], [22], [31]. The results suggest that in adult mice expression of this selected group of genes with functions related to ATE is predictive of the level of fat mass that existed at 10 days of age. If modulation of the expression of a small select group of genes is critical in determining ATE as an animal shuttles between states of low and high energy balance, it is important to know from the perspective of a common regulatory response to the nutrition whether these genes are coordinately regulated. Inspection of the complete list of ATE genes indicates that they all show significant positive correlations, but mRNA levels for cyclophilin b, which serves as a negative control, or PPARγ, the master gene of adipogenesis, showed no significant associations. A subset of 5 genes, which include *Cav1*, *Cav2*, Cavin, *Ldlr* and *Lpgat1* show especially high intra-correlations (boxed-in area of Fig. 6D).

#### A model based upon mothers and their offspring raised under standard control dietary conditions

**Figure 7:**
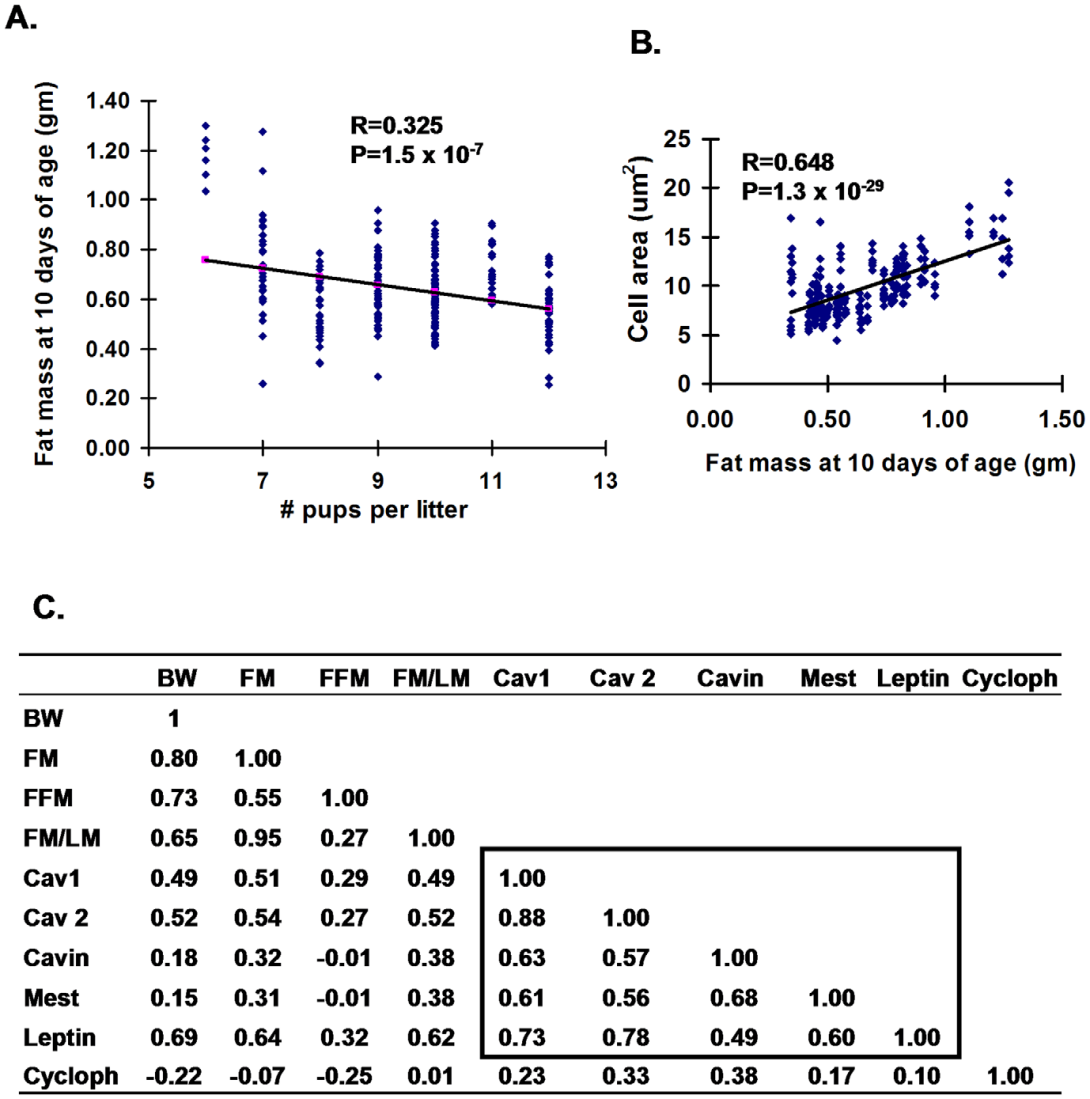
Regression analyses of adiposity and gene expression at 10 days of age. **A**. Regression analysis of litter size vs fat mass at 10 days of age (130 female and 119 male mice) indicates a weak association; **B**. the association between fat mass at 10 days of age vs cell area is very high; Average cell areas in 237 randomly chosen fields from the inguinal fat depots of 48 male mice with varying degrees of adiposity are presented. **C**. Correlation coefficients indicate strong associations between adiposity at 10 days of age and inguinal fat gene expression at 10 days of age among the ATE genes identified by microarray analysis. The 119 male mice of Cohort III were used for these analyses.

Stable variations in obesity phenotypes in adult mice fed a high fat-high sucrose diet from 8 to 16 weeks of age can emerge among mice raised under seemingly normal conditions [21]. Does a similar variation develop among B6 pups normally nursing in competition with litter-mates? At 10 days of age adiposity phenotypes were determined and inguinal fat collected for gene expression analysis and histology to determine adipocyte size (Cohort III). Since slight variations in age during the logarithmic phase of neonatal growth can cause large variation in phenotypes, 28 litters were analyzed for adiposity phenotypes. The correlation (R) between fat mass and body weight for the 249 mice was 0.76 (P = 2.2×10^−48^). The adiposity index (FM/LM; fat mass/lean mass) for 119 males ranged from 0.081 to 0.261 and for 130 females ranged from 0.064 to 0.241. There was a low, but significant correlation between litter size and fat mass (R = 0.325; Fig. 7A). Remarkably, the correlation between fat mass and adipocyte size was very high (Fig. 7B; R = 0.648) and almost identical to that observed in adult mice after diet-induced obesity, that is, a 3–4 fold range of fat mass and a 3–4 fold range in adipocyte size [22]. The expression of five genes, *Cav1* and *Cav2*, Cavin (*Ptrf*), leptin and *Mest*, with high associations to fat mass at 112 days of age also showed highly significant associations with fat mass and FM/LM at 10 days of age (Figure 7C). The data suggests that the variation in adiposity in mice raised under normal conditions during suckling is strongly associated with variation in genes of the cytoskeleton and caveolae structure. Accordingly, the associations between adiposity and expression of ATE genes in adult mice in an obesogenic environment were recapitulated in 10 day-old mice

## Discussion

The development and differentiation of adipose tissue in a mammalian organism is determined by an adipogenic processes involving the proliferation of adipocyte stem cell precursors and cellular differentiation that activates genes for the synthesis of the repertoire of adipocyte specific enzymes and structural proteins required for adipocyte function. The elucidation of this differentiation process has largely been determined through studies with 3T3-L1 cells. While many aspects of adipogenesis have been validated through studies of mice carrying target mutations to key genes of adipogenesis, the role of the nutritional environment on adipogenesis during development has been neglected. Adipogenesis as currently understood essentially describes a hard-wired differentiation program. Although there are many studies describing the effects of a high fat diet on gene expression in adult mice, the input of the environment during development in determining the capacity of adipose tissue to manage energy influx in a dynamically changing nutritional environment has not been addressed.

### The effects of the nutritional environment on gene expression during early development of adipose tissue

The complete absence of cells resembling even immature adipocytes in the newborn mouse during the first day or two of life indicates that most of adipogenesis occurs during the early post-natal period. When and where the adipoblasts or preadipocytes for the different white fat depots are formed is unknown, but currently under study. However, since adipocytes in the mouse cannot be detected histologically until after birth, their appearance coinciding with the onset of nursing underscores the fact that the development of adipose tissue in each animal occurs at the onset of a dynamically variable nutritional environment for each individual. To understand how the nutritional environment affects the capacity for adipose tissue expansion in a manner that accounts for long-term individual variability in susceptibility to diet-induced obesity, we manipulated the nutritional environment during the lactation period from birth to 21 days of age and correlated gene expression with phenotypes of adiposity and energy balance. We propose that during this early post-natal period two parallel processes determine adipose tissue development, one is the hard-wired program of the development exemplified by the appearance of transcription factors like PPARγ that activate the adipocytes biomarkers like aP2, lipoprotein lipase, and glycerol-3 phosphate dehydrogenase. This stringently controlled developmental program is largely resistant to the variable nutritional environment. Heat maps of genes from microarray analysis illustrate patterns of expression that are unaffected by the nutritional environment (Fig. 2C). However, there are other genes with expression that is very sensitive to the nutritional environment in an age-dependent manner; some genes are unaffected by a high fat diet during early development, but are highly induced by a high fat diet as adults, irrespective of their early history of malnutrition (Fig. 2A). Still other genes show variable expression in both suckling and adult mice in a manner that is dependent on the nutritional environment during the suckling period (Figs. 2B and 3B). These latter genes, particularly those shown in Fig. 3B, are not stringently controlled, rather their expression in very sensitive to the nutritional environment. We searched among these genes for determinants of variable adipose tissue expansion. There were between 500 and 750 genes with significantly different levels of expression between control and LUN groups at 5 and 10 days of age or between adult mice before (56 days of age) and after a high fat diet for 8 weeks (112 days of age) (Fig. 3C). Using VENN analysis as described in Fig. 3C we identified 53 genes that were associated with adipose tissue expansion independent of age or diet (Fig. 3C and 4). That none of these genes encode transcription factors of adipogenesis or biomarkers of adipocytes suggests that adipose tissue expansion has an additional repertoire of genes that are regulated independently of those commonly associated with adipogenesis.

### Genes of caveolae and the cytoskeleton cluster with *Mest* during adipose tissue expansion

**Figure 8:**
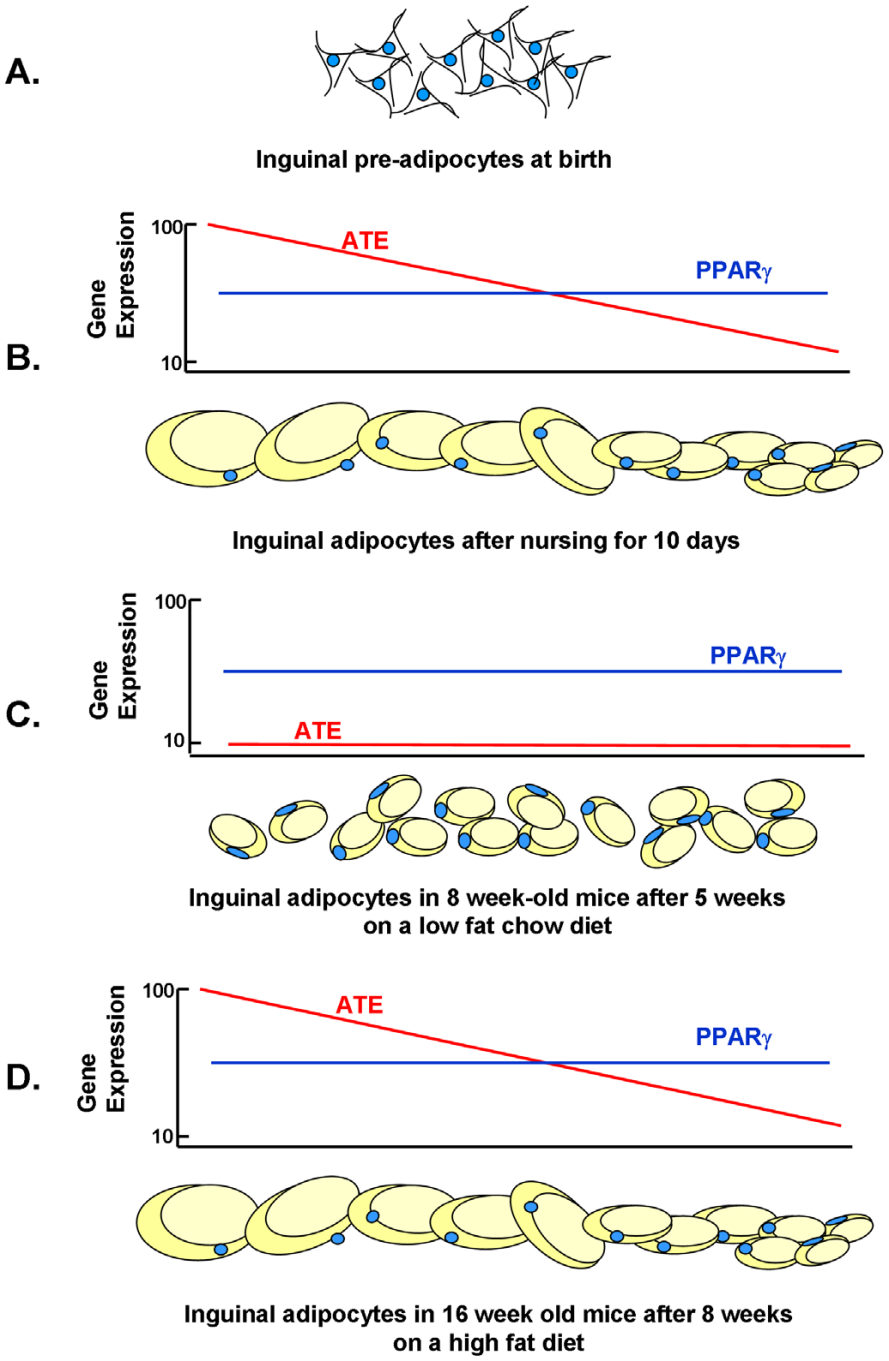
Model for the relationship between adipocyte size as determined by the nutritional environment and the expression of genes of adipose tissue expansion (ATE). **A**. At birth the absence of mature adipocytes precludes assessment of genes linked to adiposity. **B**. After 10 days of post-natal development mice raised in a control or LON environment develop adipocytes with a range of sizes that is correlated with the level of ATE genes; Expression of PPARγ has no significant relationship with adipocyte size. **C**. Following a low fat chow diet for 5 weeks from weaning, the size of adipocytes is reduced and the expression of ATE genes is strongly suppressed, while PPARγ expression is maintained at a stable level. **D**. The adipocytes expand in size when mice are fed a high-fat diet for 8 weeks and the association between adipose size and ATE gene expression is re-established.

A model of the progression of adipose tissue expansion from birth to 10 days of age when the nutrition environment causes a positive energy balance that drives changes in ATE gene expression that determine the capacity of the adipocyte to maximize fat storage is shown (Figure 8). As the mouse enters a state of positive energy balance due to consumption of fat-enriched mother's milk as a pup or a high-fat obesogenic diet as an adult, a minimum of 15 genes are coordinately induced to very high levels. Several of these genes encode proteins that through their association with caveolae structure, the cytoskeleton and lipid vesicle formation have the capacity to control the storage of excess fat associated with a positive energy balance, Others, such as *Mest* and *Bmp3*, are highly correlated with fat mass expansion, although their biochemical functions have not yet been determined. We have previously shown, through studies of adult mice exposed to the cold, that the induction of *Mest* is related to fat mass expansion and not the high fat diet [22]. We are proposing that the capacity for adipose tissue expansion begins by 5 days of age as based upon the correlations for fat mass at 5 vs 10 days of age and 10 vs 112 days of age in control (CON) or over-nourished (LON) mice, but not under-nourished (LUN) mice (Fig. 6). Furthermore, the molecular underpinning of the nutritionally determined variability in adipose expansion is the set of genes described here, corroborated also with phenotypes of the *Cav1*, *Ptrf* (Cavin1) and *Vldlr* mouse KO models (Fig. 3 and 4). The model in Figure 8 shows one cycle from post-natal programming to the contraction of the fat depot and reduced levels of gene expression when mice are fed a low fat chow diet, then followed by the response to a high fat diet as a adult. However, such cycles of adipose tissue contraction and expansion are predicted to reoccur during adult life as an individual goes through repeated cycles of food restriction and over-eating. Obviously, the mechanism for adipocyte is vastly more complex, involving the adipogenesis program, and proteins involved in the structure of lipid vesicles; however, as we have pointed out the expression of these genes, commonly considered to be adipogenic, are not variably expressed at the mRNA levels (Supplemental data Fig. S1). The possibility that transcription factors of adipogenesis are modified post-translationally to affect nuclear transport, chromatin interactions or protein degradation, thereby modulating ATE gene transcription, will be an area of future investigation.

Although microtubules and caveolins have been implicated in the insulin-dependent translocation of Glut4 in both 3T3-L1 cells and rat primary adipocytes, it has not been established how the cytoskeletal elements are important to the function of adipose tissue *in vivo* [32]–[34]. Based upon expression studies we postulate a role for caveolae and associated structures in adipose tissue that is related to the accumulation (uptake) and storage of lipid. This putative role is completely consistent with the loss of diet-induced obesity in mice with a targeted inactivation of either *Cav1* or *Ptrf* (Cavin) [35], [36]. CAV2 is also associated with caveolae structure, and *Cav2* KO mice have abnormal pulmonary function, but no apparent defect in adipocyte structure or function [37]. In addition to CAV1, CAV2 and PTRF/Cavin1; Cavin3 (SRBC/PRKCBP), but not Cavin2, are proteins integral to the structure of caveolae-associated or adapter molecules vital to maintain the concentration of caveolin proteins necessary for caveolae structure [36], [38], [39]. Evidence that downregulation of *Ptrf*/Cavin1 affects the levels of *Cav1* is consistent with the remarkable coordinate expression that occurs at the mRNA level, however, such co-ordinate expression *Ptrf*/Cavin1 and *Cav1* cannot be interpreted as a transcriptional role for *Ptrf*/Cavin1, since similar high correlations of gene expression were found among *Cav1*, *Ptrf*/Cavin1, *Cav2*, *Lpgat1* and *Vldlr* (Fig. 6D). Central to a mechanism in which activation and assembly of the caveolae structure is induced to facilitate the transport of fat from the circulation to lipid vesicles is annexin A2 which is induced and highly expressed at the time of ATE. Annexin A2 has been shown to be a key mediator in modulation of cell morphology through its interactions with the cytoskeleton and lipid rafts including actin and the caveolins [40] and to be part of a complex with caveolins for cholesteryl ester transport [41]. The combination of the caveolins and cytoskeletal elements, together with the fact that *Vldlr* [42] and *Mest* KO [22] mice also have reduced DIO, suggest a dynamic remodeling of a structure involved in facilitating the uptake and assembly of lipids into the lipid vesicles. Surprisingly, the PAT proteins, such as perilipin, while vital components of lipid vesicle structure and function did not emerge with a variable pattern of expression related to ATE according to our selection criteria [22], [43]–[45]. Since caveolae and cytoskeletal-associated genes in the adipose tissue are expressed at very high levels during early development at the time of nursing and again in the adult when in a positive energy status from being fed a high fat diet, the results suggests that the expansion of adipose tissue *in vivo* in response to energy status involves dynamic changes in caveolae and cytoskeletal structure.

### Regulating the levels of transcripts for *Mest* and genes of adipose tissue expansion

Between birth and 10 days of age active cell division is occurring in cells destined for the adipocyte lineage and certainly transcription is also active for genes required to form mature adipocytes; nevertheless, the data suggests selective increases in mRNAs for the ATE genes occur in concert with variations in the capacity for fat mass expansion. This differential read out of the adipogenesis program as a result of the nutritional environment generates variable phenotypes of the mouse that are retained at least until after the 4 month duration of this experiment and probably for the lifetime of the animal. Understanding the basis of a transcriptional mechanism that targets a selective set of genes required for adipocyte formation is an important objective, since it could provide insights into the mechanism of nutritional programming. An obvious place to look for transcription regulators is with the regulatory genes of adipogenesis which include PPARγ and the C/EBP isoforms. These genes are critical for adipogenesis based upon correlations of their expression profiles in 3T3-L1 preadipocytes with *in vitro* differentiation phenotypes and later from the adiposity phenotypes found in mice carrying targeted mutations [46]. While mutations of these genes have confirmed they are necessary for adipogenesis, it is less certain how these transcription factors are involved in determining the response of the animal to an obesogenic environment. No significant correlations were found between PPARγ2 and fat mass (Fig. 6D). The DOK2-dependent phosphorylation of serine 112 of PPARγ has been implicated in the induction of obesity by a high fat diet [47]; however, the absence of an effect of the PPARγ (S112A) on adiposity does not suggest that this is important mechanism for regulation of ATE genes in mice with variable diet-induced obesity. Nevertheless, there is evidence in tumor cells that *Cav1* and *Cav2* are up-regulated by PPARγ in human carcinoma cells [48]; annexin II is up-regulated by PPARγ agonists in 3T3-L1 cells [49] and *Cav1* is suppressed by Ras-p42 MAP kinase and PKA [50]. It should be emphasized that PPARγ and other transcription factors in ATE may be involved through post-translational mechanisms that control protein stability or nuclear localization, which have not been addressed by our studies.

### Nutritional programming of adult phenotypes during early development

Thus, the highly significant correlations of gene expression and fat mass at 10 and 112 days of age as well as high correlations between the same genes of lipid vesicle and cytoskeleton structure indicate that this is a prime system with which to investigate the basis of nutritional programming. The variable capacity to expand adipose tissue need not be associated with pathology, rather it is a normal variation in adipose tissue plasticity, occurring during both malnutrition and normal post-natal conditions that enables the animal to manage its nutritional environment (Fig. 7). The phenomenon of nutritional programming initially emerged from an epidemiological study of adult men who were at critical stages of fetal or post-natal development during the Dutch Famine in the winter of 1945 [7] and from correlations between reduced birth weights and the incidence of cardiovascular diseases [8]–[11]. Investigations on the physiological and molecular basis of nutritional programming using experimental animal models have largely focused on the role of nutritionally determined perturbations in the levels of circulating hormones, in particular glucocorticoid, insulin and leptin, in the mother during fetal development that may affect development of the pancreas, kidney and hypothalamus, etc. [51]. Stress-induced glucocorticoids in the fetus have been implicated in the development of hypertension, hyperglycemia, and behavioral abnormalities [52] and it has been postulated that over-nutrition during the pre-weaning period of rats increases diet-induced obesity in adults through an adipose tissue specific glucocorticoid-mediated pathway [53]. Our over-nutrition protocol did not produce a significant increase in adiposity compared to controls nor any consistent changes in 11ß hydroxysteroid dehydrogenase I mRNA levels in either post-natal or adult inguinal fat (data not shown). Of considerable interest is the rat pup-in-a-cup model for over-nutrition during the post-natal period which shows persistence of hyperinsulinemia in adults and increased expression of lipogenic enzymes in liver and adipose tissue that is probably specific to the high carbohydrate diet fed to the animals [54].

### Suppression of leptin during early under-nutrition does not affect food intake

The power of the mouse model for studies of nutritional programming is that development of white adipose tissue occurs exclusively during the post-natal period, thereby coinciding with the onset of nursing. The subcutaneous inguinal fat first appears between day 2 and 5 and then on about day 9 the visceral fat pad emerges. Therefore, one can ascertain, as shown in Fig. 1C, that the levels of plasma leptin are almost completely determined by leptin secreted by the inguinal fat (correlation coefficient, R, between plasma leptin and leptin mRNA in inguinal fat equals 0.89). While it is known that maternal under-nutrition or protein deficiency leads to underweight pups at birth and a shift in the leptin surge, the consequences of under- and over-nutrition on circulating leptin during the post-natal period have not been previously been described. Compared to the control group, over-nutrition does not have significant effects on circulating leptin levels (Fig. 1C and Table 1); however, under-nutrition severely suppressed the level of plasma leptin (Fig. 1C). In a group of 24 mice in the under-nutrition protocol, only 5 showed leptin quantifiable by an ELISA assay. However, despite this severe suppression of circulating leptin and both reduction in lean and fat mass due to under-nutrition during the lactation period, weaned mice that were fed a chow diet *ad libitum* until 8 weeks of age and then a high fat diet for another 8 weeks, showed no affects of this suppression of leptin on food intake (Table 1). In *ob/ob* mouse completely lacking functional leptin, an attenuation in the development of the neuronal feeding circuitry in the hypothalamus has been proposed to be the basis for their hyperphagic phenotype [55]. It was also striking that over-nutrition (high fat diet and small litter size to reduce competition) did not increase susceptibility to DIO, even though it was clear from the microarray data that profound, widespread changes in gene expression were caused by the high fat diet fed to the dam (Fig. 2D). Accordingly, feeding a high fat diet to the mother under conditions that promote over-feeding by the pups did not increase obesity compared to controls. As recently reviewed [56], perturbations in long term susceptibility to obesity have been shown in several studies, but these have generally been based upon models of gestational nutritional manipulation, i.e. low protein diets that affect beta cells and vascularization in the developing pancreas [57], [58] and hypocaloric diets [14], [26], [59]. Accordingly, any effects on food intake potentially involves pancreatic endocrine factors as well as leptin secretion[60]; however, in the study described herein with a total of 325 mice in 2 separate experiments, over- and under-nutrition during the post-natal period generated huge effects on circulating insulin and leptin, but no long term effects on food intake. It is also evident that the over-nutrition protocol, in particular the 58 Kcal% fat diet fed to the mother, while having profound effects on gene expression (Fig. 2D), had only small, transient effects on body weight compared to the control mice and no effects on food intake (Table 1).

### Summary

In summary, we have identified a set of genes, which we have called adipose tissue expansion (ATE) genes. These genes are up-regulated when adipocytes are accumulating fat and expanding during early development and in adult mice fed a high fat diet in a manner consistent with nutritional programming. The ATE genes encode proteins associated with caveolae and other cytoskeletal structures of the adipocyte that are involved in the assembly of lipid vesicles in a positive energy state. Inactivation of several of these genes by gene targeting has shown that loss of function reduces the capacity of the mutant mouse for diet-induced obesity, but adipogenesis *per se* appears normal. Correlation analyses of gene expression patterns and adiposity phenotypes indicates that the ATE genes are stringently co-regulated and that expression patterns established by the nutritional environment during suckling are retained in the adult animal. Finally, this model of over and under-nutrition from birth until weaning causes large changes in circulating insulin and leptin levels, but no long-term effects on food intake.

## Materials and Methods

### Animals

C57BL/6J breeders were obtained from the Jackson Laboratory (Bar Harbor, Maine, United States) and maintained at Pennington Biomedical Research Center as described [21]. Newborn mice were raised from birth to weaning with one of three sets of nutritional conditions (Fig. 1D): 1.) the control condition had 8 pups per litter and the mother was fed the breeder diet 5015 (22 kcal % fat) *ad libitum*. 2.) Lactation under-nutrition condition (LUN) had 8 pups per litter, but the mother was only fed 50% of the food (LabDiet 5015) consumed by the control mice the previous 24 hr. 3.) Lactation over-nutrition condition (LON) had litter size reduced to 4 pups per litter and the mother was fed a high fat 58 Kcal % fat diet (Research Diet 12331, New Brunswick, New Jersey, United States) *ad libitum*. After weaning the offspring from the 3 nutritional conditions were treated the same; from weaning until 8 wk of age mice were fed a low fat chow diet (LabDiets 5053 11 Kcal % fat) *ad libitum*. At 8 wk of age mice were fed *ad libitum* a high saturated fat diet D12331 (Research Diets,) for 8 weeks. From weaning until 7 wk of age male mice were group housed (3–5 mice per pen) until 7 wk of age, at which time they were singly housed for the remainder of the experiment. Cohort 3 comprised 249 total mice (119 male mice) raised from birth until 10 days of age with mothers fed the standard breeder Labdiet (5015); litter sizes ranged from 6 to 12 pups. All protocols have been approved by the Pennington Biomedical Research Center's Institutional Animal Care and Use Committee.

### Phenotyping

Adiposity was determined from body weights and measurements of body composition by nuclear magnetic resonance (NMR, Bruker, The Woodlands, Texas, United States). Inguinal adipose tissue from 10 day old mice was fixed in formalin, embedded in paraffin; 10_µm sections were stained with eosin/hematoxylin and photographed at constant settings. Adipocyte cell size, food intake, RNA isolaton and qRT-PCR was determined as described [22], [61]. Standard curves were generated using pooled RNA from individual samples within each experiment. All samples were analyzed in duplicate and expression levels were calculated relative to input of total RNA. This method of calculating gene expression was preferred over normalization to a housekeeping gene, because such genes are not constant under the broad range developmental and nutritional conditions of our experimental protocols. Cyclophilin B was included as a control gene in our analyses. Probe and primer sequences used to perform the analyses are available upon request. Plasma was collected for analysis of leptin and insulin per the instruction of the suppliers (Linco Inc. St. Charles, Missouri). Western blots for α-tubulin and annexin IIa were performed as described previously [22].

### Microarray analysis

Gene expression profiles were generated using Applied Biosystems Mouse Genome Survey Microarray as previously described [21]. Each microarray contains approximately 34,000 features that include a set of about 1,000 features that were used for quality control checks for IVT labeling, RT labeling, hybridization, dynamic range of chemiluminescent detection, spatial calibration, cross-talk and assay background. Each microarray uses 32,996 probes to interrogate 32,381 curated genes representing 44,498 transcripts. Signal intensities across all microarrays were normalized using the quantile-quantile method (www.bioconductor.org) to provide consistency of signal strength across developmental and dietary conditions. It was determined from our previous microarray analysis of gene expression in DIO B6 mice that the application of false discovery rate (FDR) corrections caused a failure in the ability to identify genes associated with variations in DIO that can be readily validated via qRT-PCR [21]. We have applied the Benjamini-Hochberg FDR correction with microarrays generated in this study and have shown that regardless of which arrays were compared to detect differences in gene expression, relatively few genes showed a significance of ≤0.05 using an FDR adjusted P-value [62]. This is likely caused by the increased variance caused by the large linear dynamic range (>10,000-fold in our experiments) and increased sensitivity of the ABI1700 chemiluminescent system compared to other microarray platforms. Therefore, genes were selected based on significance from 2-tailed T-Tests between replicates of each comparison. In support of our established methods in identifying differentially expressed genes, supplemental Figure S2 shows high similarity between quantile-quantile normalized microarray expression data and gene expression measured by qRT-PCR across developmental time and dietary conditions. K-means and Venn analyses as described in the research results were conducted with Spotfire DecisionSite Software (Spotfire Inc., Somerville, Massachusetts),

Microarray experiments, described according to MIAME guidelines, have been deposited in the GEO repository. The accession number is GSE19809.

### Statistical analysis

The significance of differences among control, over-nutrition and under-nutrition groups in Fig. 1B and Table 1 were first determined by single factor ANOVA, followed by post-hoc 2-tailed T-Tests to establish significant differences between groups. Correlations of morphological and gene expression phenotypes as a function of age were determined by regression analysis. A correlation coefficient greater than 0.3 was considered significant at P = 0.01.

## Supporting Information

Figure S1 Expression of RNA for transcription factors of adipogenesis during development and in adult mice exposed to diet-induced obesity.
(0.19 MB DOC)

Figure S2 Validation of gene expression levels from microarray data by quantitative RT-PCR using TaqMan probes as described in the Materials and Methods.
(0.57 MB DOC)

Table S1 Genes selected by Venn analysis for association with adipose tissue expansion.
(0.06 MB DOC)
