# Supplementary material for: The Early Nutritional Environment of Mice Determines the Capacity for Adipose Tissue Expansion by Modulating Genes of Caveolae Structure
Source: PLoS One. 2010 Jun 21;5(6):e11015. doi: 10.1371/journal.pone.0011015 (PMC2888576; doi:10.1371/journal.pone.0011015)
Supplement: Table S1 — Genes selected by Venn analysis for association with adipose tissue expansion. (0.06 MB DOC) [file pone.0011015.s003.doc]

**Table S1**

**Genes selected by Venn analysis for association with adipose tissue expansion**

| | **Gene_Symbol** | **Gene_Name** | | --- | --- | | Anxa2 | annexin A2 | | AW112037 | expressed sequence AW112037 | | B430320C24Rik | RIKEN cDNA B430320C24 gene | | BC004044 | cDNA sequence BC004044 | | Bgn | biglycan | | Bmp3 | bone morphogenetic protein 3 | | C730029A08Rik | RIKEN cDNA C730029A08 gene | | Cav1 | caveolin, caveolae protein 1 | | Cav2 | caveolin 2 | | Cdkn2b | cyclin-dependent kinase inhibitor 2B (p15, inhibits CDK4) | | Crtac1 | cartilage acidic protein 1 | | Emp1 | epithelial membrane protein 1 | | Fads3 | fatty acid desaturase 3 | | Flnc|LOC545833 | filamin C, gamma (actin binding protein 280) | | Flot1 | flotillin 1 | | Fzd4 | frizzled homolog 4 (Drosophila) | | Hist2h3c2 | histone 2, H3c2 | | Inhbb | inhibin beta-B | | Insig1|LOC625787 | insulin induced gene 1 | | Lctl | lactase-like | | Ldlr | low density lipoprotein receptor | | Lep | leptin | | Lhfpl2 | lipoma HMGIC fusion partner-like 2 | | Lpgat1 | lysophosphatidylglycerol acyltransferase 1 | | Mest | mesoderm specific transcript | | Myadm | myeloid-associated differentiation marker | | Npr3 | natriuretic peptide receptor 3 | | Olfm1 | olfactomedin 1 | | Palmd | palmdelphin | | Pcbd1 | pterin 4 alpha carbinolamine dehydratase/ | | Pkp2 | plakophilin 2 | | Prkcdbp | protein kinase C, delta binding protein | | Ptrf | polymerase I and transcript release factor | | Rarres2 | retinoic acid receptor responder (tazarotene induced) 2 | | Rasd1 | RAS, dexamethasone-induced 1 | | Rnd3 | Rho family GTPase 3 | | Rp1hl1 | retinitis pigmentosa 1 homolog (human)-like 1 | | Rtn4rl1 | reticulon 4 receptor-like 1 | | Scd1 | stearoyl-Coenzyme A desaturase 1 | | Scd2 | stearoyl-Coenzyme A desaturase 2 | | Serpina3n | serine (or cysteine) peptidase inhibitor, clade A, member 3N | | Slc16a12 | solute carrier family 16 member 12 | | Sncb | synuclein, beta | | Sncg | synuclein, gamma | | Tmem45b | transmembrane protein 45b | | Trp53inp2 | tumor protein p53 inducible nuclear protein 2 | | Tspan17 | tetraspanin 17 | | Tuba1 | tubulin, alpha 1 | | Tuba2| |Tuba6| | tubulin, alpha 2|tubulin, alpha 6 | | Tuba8 | tubulin, alpha 8 | |
| --- | --- | --- | --- | --- | --- | --- | --- | --- | --- | --- | --- | --- | --- | --- | --- | --- | --- | --- | --- | --- | --- | --- | --- | --- | --- | --- | --- | --- | --- | --- | --- | --- | --- | --- | --- | --- | --- | --- | --- | --- | --- | --- | --- | --- | --- | --- | --- | --- | --- | --- | --- | --- | --- | --- | --- | --- | --- | --- | --- | --- | --- | --- | --- | --- | --- | --- | --- | --- | --- | --- | --- | --- | --- | --- | --- | --- | --- | --- | --- | --- | --- | --- | --- | --- | --- | --- | --- | --- | --- | --- | --- | --- | --- | --- | --- | --- | --- | --- | --- | --- | --- | --- |
