# Supplementary material for: The Early Nutritional Environment of Mice Determines the Capacity for Adipose Tissue Expansion by Modulating Genes of Caveolae Structure
Source: PLoS One. 2010 Jun 21;5(6):e11015. doi: 10.1371/journal.pone.0011015 (PMC2888576; doi:10.1371/journal.pone.0011015)
Supplement: Supplementary file 2 [file pone.0011015.s001.doc]

Figure S1

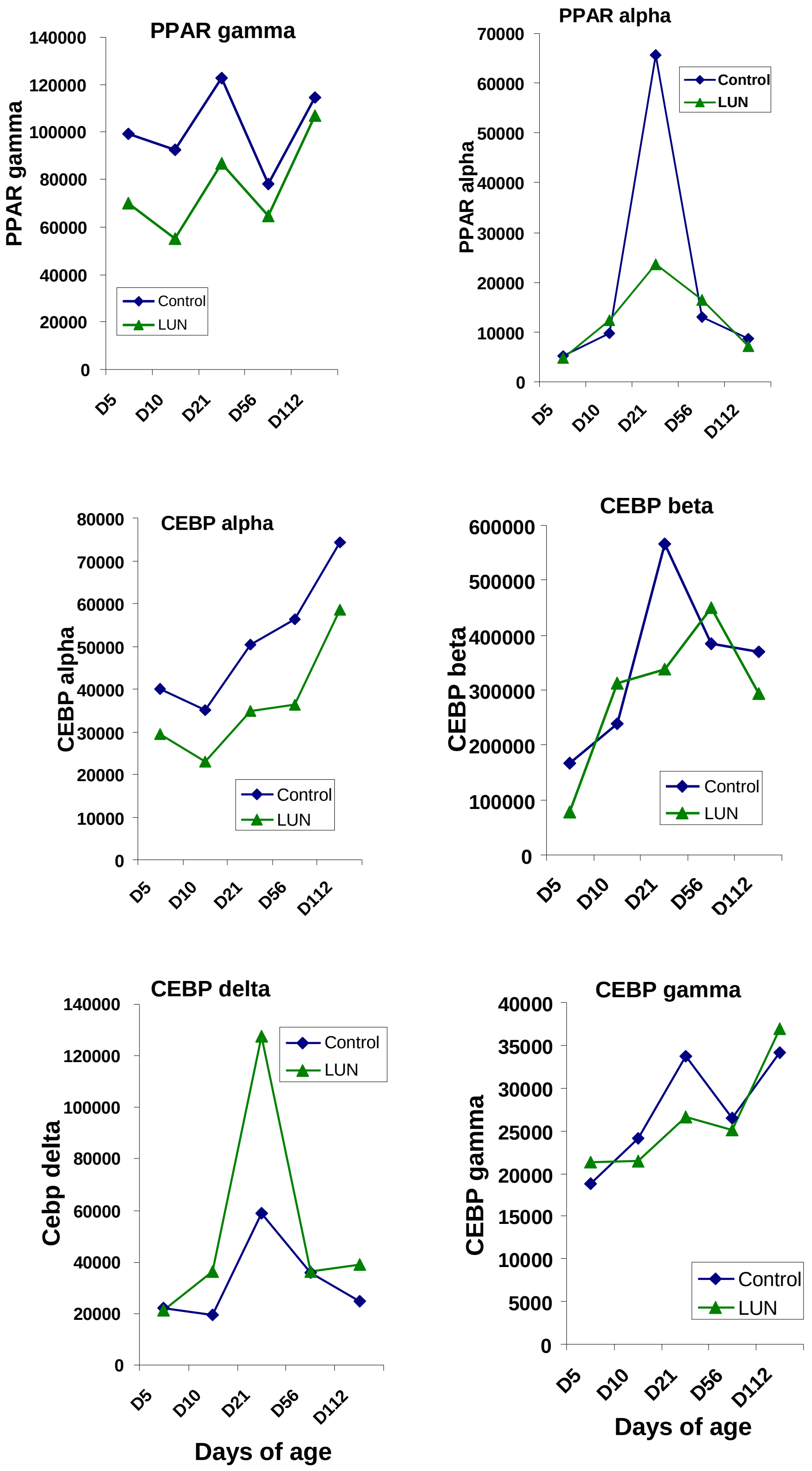
PPAR gamma
PPAR gamma
140000
120000
100000
80000
60000
40000
20000
0
Control
LUN
D5
D10
D21
D56
D112
PPAR alpha
PPAR alpha
70000
60000
50000
40000
30000
20000
10000
0
Control
LUN
D5
D10
D21
D56
D112
CEBP alpha
CEBP alpha
80000
70000
60000
50000
40000
30000
20000
10000
0
Control
LUN
D5
D10
D21
D56
D112
CEBP beta
CEBP beta
600000
500000
400000
300000
200000
100000
0
Control
LUN
D5
D10
D21
D56
D112
CEBP delta
Cebp delta
140000
120000
100000
80000
60000
40000
20000
0
Control
LUN
D5
D10
D21
D56
D112
Days of age
CEBP gamma
CEBP gamma
40000
35000
30000
25000
20000
15000
10000
5000
0
Control
LUN
D5
D10
D21
D56
D112
Days of age
