# Supplementary material for: The Early Nutritional Environment of Mice Determines the Capacity for Adipose Tissue Expansion by Modulating Genes of Caveolae Structure
Source: PLoS One. 2010 Jun 21;5(6):e11015. doi: 10.1371/journal.pone.0011015 (PMC2888576; doi:10.1371/journal.pone.0011015)
Supplement: Supplementary file 3 [file pone.0011015.s002.doc]

Figure S2

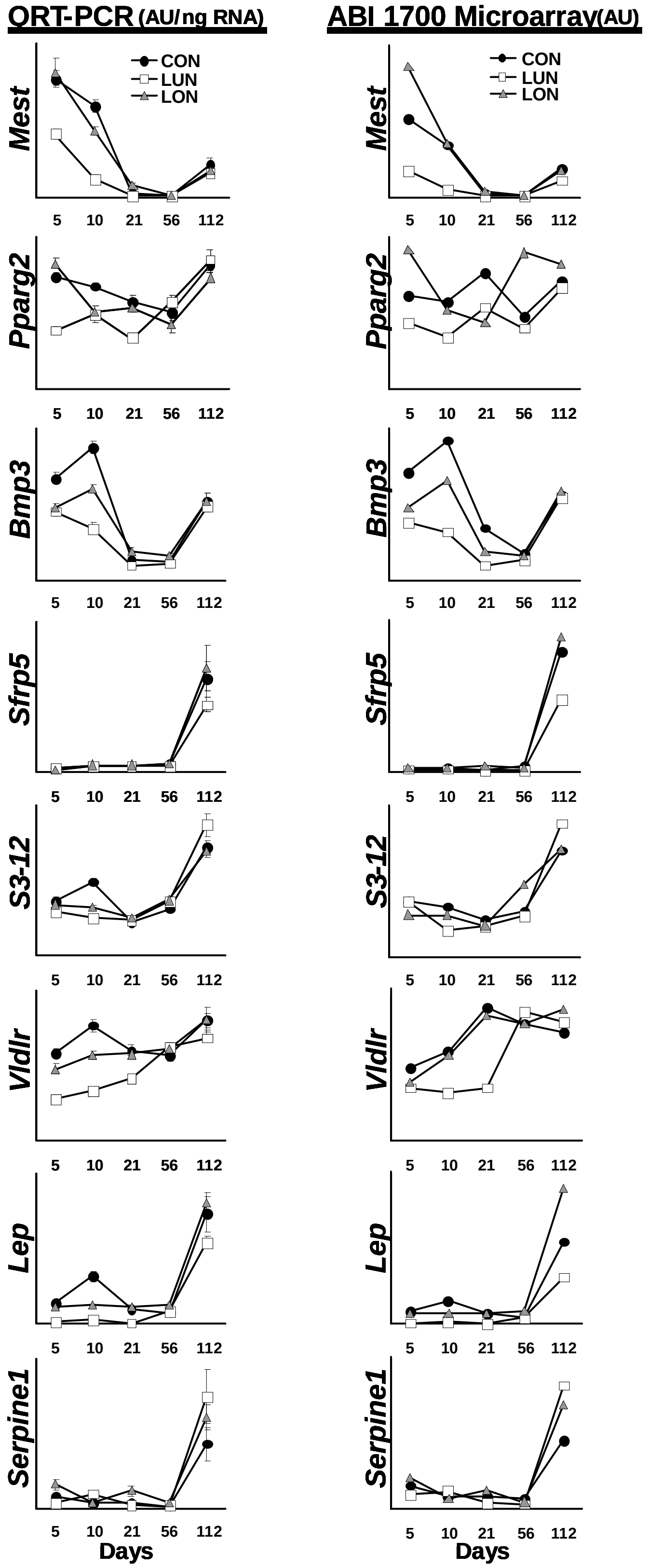
QRT-PCR (AU/ng RNA)
ABI 1700 Microarray (AU)
CON
LUN
LON
Mest
Pparg2
Bmp3
Sfrp5
S3-12
Vldlr
Lep
Serpine1
5 10 21 56 112
Days
